# Supplementary material for: MetaRibo-Seq measures translation in microbiomes
Source: Nat Commun. 2020 Jun 29;11:3268. doi: 10.1038/s41467-020-17081-z (PMC7324362; doi:10.1038/s41467-020-17081-z)
Supplement: Supplementary file 10 — Supplementary Data 7 [file 41467_2020_17081_MOESM10_ESM.zip › File2/Confidence_High_Taxonomy/201602_out.krona.html]

Javascript must be enabled to view this page.

members
magnitude
magnitudeUnassigned
count
unassigned
taxon
rank

201602\_out

7

superkingdom
6
2759

4751
kingdom
1

451864
subkingdom
1

phylum
1
4890

subphylum
1
147538

147550
1
class

222543
1
subclass

order
1
5125

5129
1
family

1
genus
5543

SRS053356\_contig\_number\_33171

5
kingdom
33208

phylum
5
7711

89593
subphylum
5

8287
superclass
5

class
5
40674

superorder
5
314146

order
5
9443

suborder
5
376913

infraorder
5
314293

parvorder
5
9526

5
superfamily
314295

5
family
9604

207598
subfamily
5

5
genus
9605

9606
5
species

SRS013687\_contig\_number\_contig-100\_4026.157469SRS023914\_contig\_number\_27456SRS051031\_contig\_number\_contig-100\_324.178228SRS076929\_contig\_number\_24018SRS1041145\_contig\_number\_30433

superkingdom
1
2

1
phylum
1117

order
1
1890424

family
1
1890438

genus
1
1005722

268141
species
1

SRS098571\_contig\_number\_63498
